# The Action of Di-(2-Ethylhexyl) Phthalate (DEHP) in Mouse Cerebral Cells Involves an Impairment in Aryl Hydrocarbon Receptor (AhR) Signaling

**DOI:** 10.1007/s12640-018-9946-7

**Published:** 2018-08-18

**Authors:** Anna K. Wójtowicz, Agnieszka M. Sitarz-Głownia, Małgorzata Szczęsna, Konrad A. Szychowski

**Affiliations:** 1 Department of Animal Biotechnology, Animal Sciences Faculty, University of Agriculture, Redzina 1B, 30-248 Krakow, Poland; 2 Department of Clinical Biochemistry, University of Opole, kard. B. Kominka 6a, 45-032 Opole, Poland

**Keywords:** DEHP, AhR, Cyp1a1, Glia, Neurons, ROS

## Abstract

Di-(2-ethylhexyl) phthalate (DEHP) is used as a plasticizer in various plastic compounds, such as polyvinyl chloride (PVC), and products including baby toys, packaging films and sheets, medical tubing, and blood storage bags. Epidemiological data suggest that phthalates increase the risk of the nervous system disorders; however, the impact of DEHP on the brain cells and the mechanisms of its action have not been clarified. The aim of the present study was to investigate the effects of DEHP on production of reactive oxygen species (ROS) and aryl hydrocarbon receptor (AhR), as well as Cyp1a1 and Cyp1b1 mRNA and protein expression in primary mouse cortical neurons and glial cells in the in vitro mono-cultures. Our experiments showed that DEHP stimulated ROS production in both types of mouse neocortical cells. Moreover, the results strongly support involvement of the AhR/Cyp1A1 signaling pathway in the action of DEHP in neurons and glial cells. However, the effects of DEHP acting on the AhR signaling pathways in these two types of neocortical cells were different. In neurons, *AhR* mRNA expression did not change, but AhR protein expression decreased in response to DEHP. A similar trend was observed for Cyp1a1 and *Cyp1b1* mRNA and protein expression. Failure to induce Cyp1a1 in neurons was confirmed by EROD assay. In primary glial cells, a decrease in AhR protein level was accompanied by a decrease in *AhR* mRNA expression. In glial cells, mRNA and protein expression of Cyp1a1 as well as Cyp1a1-related EROD activity were significantly increased. As for Cyp1b1, both in neurons and glial cells *Cyp1b1* mRNA expression did not significantly change, whereas Cyp1b1 protein level were decreased. We postulate that developmental exposure to DEHP which dysregulates AhR/Cyp1a1 may disrupt defense processes in brain neocortical cells that could increase their susceptibility to environmental toxins.

## Introduction

Di-(2-ethylhexyl) phthalate (DEHP) is used as a plasticizer in various plastic compounds such as polyvinyl chloride (PVC), and products including toys, food packaging film and sheets, medical devices, and blood storage bags and household products (Tickner et al. 2001; Szychowski and Wójtowicz 2013). Due to the unbound nature of the polymer, DEHP can easily leach from products (Pearson and Trissel 1993). DEHP pollutes the environment and is detected in samples from soil, indoor air, water, plants, and human foods (Tran et al. 2017; Wowkonowicz and Kijeńska 2017). For the human population, the main source of DEHP for can be found in contaminated food with which DEHP comes into contact during the production process (Fierens et al. 2012; Heinemeyer et al. 2013). DEHP and its metabolite, mono-(2-ethylhexyl)-phthalate (MEHP), can be detected in human tissues and bodily fluids, such as amniotic fluid, blood, milk, or urine (Silva et al. 2004; Sakhi et al. 2017). After single oral application of 500 μM/kg DEHP to marmosets, high concentration of DEHP maintain in blood by approximately 6 h (Rhodes et al. 1986). However, we should remember that people have chronic contact with this compound through lifetime. Typical human exposure is estimated to be 4–30 μg DEHP kg^−1^ day^−1^, but some individuals have substantially greater exposure resulting from different DEHP-plasticized medical devices (Doull et al. 1999; Moore et al. 2001). DEHP and MEHP have been reported to easily pass through biological barriers, such as the placental barrier or blood-brain barrier, and can affect development and proper nervous system function (Shin et al. 2014; Lin et al. 2015a; Komada et al. 2016). DEHP levels were 1.15 ± 0.81 μg mL^−1^ in maternal plasma and 2.05 ± 1.47 μg mL^−1^ in the cord plasma (Tanida et al. 2009; Lin et al. 2015a). To date, in utero exposure to DEHP (1500 mg kg^−1^) was found to cause metabolic disturbance of lipid metabolome in the fetal brain (Xu et al. 2007). Moreover, DEHP exposure prenatally has been demonstrated to affect neurons in the sexual differentiation area of rat brains and subsequently lead to neurodegeneration (Moore et al. 2001; Dhanya et al. 2003). Furthermore, postnatal exposure to DEHP causes motor hyperactivity and a strongly reduced number of dopaminergic neurons (Masuo et al. 2004; Tanida et al. 2009). Although there is an increasing body of evidence that shows the deleterious effects of DEHP on the nervous system, little is known about its mechanism of action on mammalian cerebral cells.

Aryl hydrocarbon receptor (AhR) is a ligand activated transcription factor and is a nuclear xenobiotic receptor that plays a crucial role in cellular cytochrome expression (Beischlag et al. 2008; Lindsey and Papoutsakis 2012). In addition, activation of AhR inhibits cells from differentiating into astrocytes but promotes differentiation into neurons (Takanaga et al. 2004; Akahoshi et al. 2006). The main genes that AhR targets are the cytochrome P450 enzymes (CYP), such as Cyp1a1 and Cyp1b1 (Guengerich et al. 2003). Cyp1a1 and Cyp1b1 are responsible for the metabolism of hydrophobic polycyclic aromatic hydrocarbons (PAHs) and polyhalogenated aromatic hydrocarbons (PHAHs), such as dioxin-like compounds and polychlorinated biphenyls (PCBs) (Nebert et al. 2004; Nebert and Dalton 2006). However, the role of AhR signaling in the response of cerebral cells to DEHP has not been reported.

DEHP has been reported to induce *Ahr* and *Cyp1b1* mRNA in the cerebellum of *Coturnix japonica* (quail) (Du et al. 2017). AhR activation increased the production of reactive oxygen species (ROS) due to a decrease in superoxide dismutase (SOD) activity and/or an increase in Cyp1a1 activity (He et al. 2013; Szychowski et al. 2016). ROS are known to damage lipids, proteins and DNA, which ultimately leads to apoptotic or necrotic cell death (Mittler 2017). However, the elevated ROS level is also a signaling pathway that is necessary for maintaining certain physiological processes (Schieber and Chandel 2014). In *Caenorhabditis elegans,* DEHP is able to induce toxicity and affect locomotive and thermotactic behaviors through oxidative stress (Tseng et al. 2013).

Recently, Wu et al. (2014) reported that 1 nM DEHP significantly increased ROS production in neuron-astrocyte co-cultures isolated from Balb/c mice and postulated what the cell-dependent effects were (Wu et al. 2014). Because of the interactions between ROS and AhR signaling in neuronal cells (Szychowski et al. 2016), the present study aimed to investigate the effects of DEHP on ROS production; AhR, Cyp1a1 and Cyp1b1 mRNA, and protein expression; and Cyp1a1-related EROD activity in mouse cortical neurons and glial cells in vitro.

## Materials and Methods

### Reagents

DMEM/F12 without phenol red (D2906), trypsin (T8003), charcoal/dextran-treated fetal bovine serum (FBS) (F6765), penicillin-streptomycin (P4333), l-glutamine (G3126), glycerol (G5516), Trizma base (T1503), HEPES (H3375), CHAPS (C9426), dithiothreitol (DTT) (D0632), Nonidet NP-40 (21–3277), sodium dodecyl sulfate (SDS) (L3771), 2,3,7,8-tetrachlorodibenzo-p-dioxin (TCDD) (CRM981), EDTA (798681), Tween 20 (P1379), 2′,7′-dichlorodihydrofluorescein diacetate (H_2_DCFDA) (D6883), bromophenol blue (B0126), staurosporine (S5921), phosphatebuffered saline (PBS) (P5368), DEHP (67261), an anti-β-actin antibody (A2066), and dimethyl sulfoxide (DMSO) (D2650) were purchased from Sigma–Aldrich (St. Louis, MO, USA). B27 without antioxidants (B27-AO), serum-free supplement (10889-038), neurobasal-A (12349-015) without phenol red and TaqMan probes corresponding to specific genes encoding for *Gapdh* (Mm99999915_g1), *Ahr* (Mm01291777_m1), *Cyp1a1* (Mm00487218_m1), and *Cyp1b1* (Mm00487229_m1) were purchased from Thermo Fisher Scientific (Forest City, CA, USA). The substrate for caspase-3 (235400) was purchased from Merck (Darmstadt, Germany). The cytotoxicity detection kit (LDH) (11644793001) was purchased from Roche Applied Science (Mannheim, Germany). Anti-AhR antibody, anti-Cyp1a1 antibody, anti-Cyp1b1 antibody, and Luminol Reagent (sc-8088, sc-9828, sc-32882, and sc-2048, respectively) were purchased from Santa Cruz Biotechnology, Inc. (Santa Cruz, CA, USA). Reagents for measuring protein concentration using the BioRad Protein Assay (5000006) were purchased from BioRad Laboratories (Munich, Germany). Stock solutions of these test compounds were prepared in DMSO and were added to neurobasal or DMEM/F12 medium. The final concentration of DMSO in the culture medium was always 0.1%.

### Cell Culture Preparation

Experiments were performed on cultured mouse neurons and glial cells. The cell cultures were prepared from the embryos of 15 pregnant female Swiss mice. Brain tissues were collected from mouse embryos on day 17/18 of gestation. Pregnant females were anesthetized with CO_2_ vapor and killed by cervical dislocation. Animal care followed official governmental guidelines, and all efforts were made to minimize the number and suffering of animals used. All procedures were performed in accordance with the National Institutes of Health Guidelines for the Care and Use of Laboratory Animals and were approved by the Bioethics Commission (No. 83/2012), as compliant with Polish law. Brains were removed from the embryos, and the cortical tissues were dissected. The dissected tissues were minced into small pieces and then gently digested with trypsin.

#### Neuronal Cell Culture

After tissue digestion, the cells were centrifuged, and the pellet was suspended in phenol red-free neurobasal medium supplemented with 5% charcoal/dextran-treated FBS and B2-AO supplement. The cells were plated onto poly-l-ornithine-coated (0.01 mg mL^−1^) multi-well plates. Two days after plating, the culture medium was changed to a neurobasal medium supplemented with B27-AO (2 μL mL^−1^), glutamine (2 mM), 10 U mL^−1^ penicillin, and 0.01 mg mL^−1^ streptomycin, which is recommended for primary neuronal cultures (Brewer 1995; Kajta et al. 2005). The cells were cultured at a density 1.8 × 10^5^ cells/cm^2^ for experimental purposes. This procedure typically yields cultures that contain approximately 90% neurons and 10% glial cells (Brewer et al. 1993; Brewer 1995). The cultures were maintained at 37 °C in a humidified incubator containing 5% CO_2_ and were allowed to grow for 7 days prior to the experiment. After the experiment, the culture medium was changed before the cultures were treated with the selected compounds.

#### Glial Cell Culture

After tissue digestion, the cells were centrifuged, and the pellet was suspended in phenol red-free DMEM/F12 medium supplemented with 10% fetal bovine serum (FBS), 2 mM glutamine, 100 U mL^−1^ penicillin, 0.10 mg/mL streptomycin and 250 ng/mL amphotericin B. This modified a previously described method (Wang et al. 1998; Blomstrand and Giaume 2006; Vitvitsky et al. 2006), and there are different glial culture media and techniques reviewed by Saura (2007). The cells were seeded at a density of 20 × 10^6^ cells/75 cm^2^ in culture flasks. Cultured glial cells were maintained at 37 °C in a humidified atmosphere containing 5% CO_2_. After one passage, cells that were in the logarithmic phase were collected for subsequent experiments. This technique provides a culture that is almost purely glial cells, culture contained > 90% astrocyte cell culture without any neurons (Wang et al. 1998; Blomstrand and Giaume 2006; Vitvitsky et al. 2006; Saura 2007). Cells were trypsinized with 0.25% trypsin/0.05% EDTA and passaged onto the experimental plates. The culture medium was changed prior to treating cells with the selected compound. Our isolation and culture method of cortical glial cells, resulted in an astrocyte purity of greater than 98%, was revealed using the antibody against the GFAP protein immunofluorescent staining (Szychowski et al. 2018; Electronic suplementary data).

### Measurement of Reactive Oxygen Species Production

The fluorogenic dye H_2_DCFDA was used to detect intracellular reactive oxygen species (ROS). After diffusion into the cell, H_2_DCFDA is deacetylated by cellular esterases into a non-fluorescent compound that is subsequently oxidized by ROS into 2′,7′-dichlorofluorescein (DCF) (Gomes et al. 2005). To measure the generation of ROS, the cells were seeded onto black-sided, clear-bottomed, 96-well plates in densities described above then exposed to DEHP. Five micromolars of H_2_DCFDA was applied to determine DEHP’s ability to induce ROS production in neurons and glial cells. The cells were incubated in H_2_DCFDA in serum-free and phenol red-free medium for 45 min before DEHP treatment. After 1, 3, 6, and 24 h of incubating the cells with DEHP (5% CO_2_ at 37 °C), DCF fluorescence have been measured. The interaction between DEHP and H_2_DCFDA was tested in a cell-free condition before any experiments took place, according to concerns previously described by Szychowski and Wójtowicz (2016). Hydrogen peroxide (H_2_O_2_) was used as a positive control (data not shown). DCF fluorescence was detected using a microplate reader (FilterMax F5) at maximum excitation and emission spectra of 485 and 535 nm, respectively.

### Ethoxyresorufin-*O*-Deethylase Assay

Activity of the Cyp1a1 enzyme was analyzed using the fluorometric ethoxyresorufin-*O*-deethylase (EROD) assay. The fluorescent EROD assay for Cyp1a1 activity was performed in 6-well plates according to the method described by Kennedy et al. (1993). The total protein concentration in each well was measured using fluorescamine according to the method described by Kennedy and Jones (1994). The measurement of Cyp1a1 activity was performed after 24 and 48 h of exposure to 1 to 100 nM and 1 to 100 μM DEHP or TCDD as a positive control. The EROD assays were carried out in multiwell plates, and the fluorescent product, resorufin, and the total amount of protein were quantified within the same wells using a fluorescence plate reader (Bio-Tek Instruments, Biokom). The ethoxyresorufin metabolite, resorufin, was measured using an excitation wavelength of 530 nm and an emission wavelength of 590 nm. Protein concentrations were measured using fluorescamine at an excitation wavelength of 400 nm and an emission wavelength of 460 nm.

### Real-Time PCR Analysis of mRNA

Cells were seeded onto 6-well plates to be used for real-time PCR. After 3 or 6 h of exposure to 10 μM DEHP, samples were collected and total RNA was extracted from neocortical neurons using a Qiagen RNeasy mini kit according to the manufacturer’s protocol based on the previously described method (Kajta et al. 2014). The quantity of RNA was determined using a spectrophotometer at 260 and 280 nm (ND/1000 UV/Vis; Thermo Fisher NanoDrop, USA). Two-step real-time reverse transcription (RT)-PCR was conducted. Both the RT reaction and quantitative polymerase chain reaction (qPCR) were run in a CFX96 Real-Time System (BioRad, USA). The RT reaction was performed at a final volume of 20 μL with 300 ng of RNA (as a cDNA template) using a cDNA reverse transcription kit according to the manufacturer’s protocol. Products of the RT reaction were amplified using a TaqMan Gene Expression Master Mix (Life Technologies Applied Biosystems, USA) kit using the TaqMan probes as primers for the specific genes coding for *Gadph*, *Ahr*, *Cyp1a1*, and *Cyp1b1*. Amplification was performed with a total mixture volume of 20 μL containing 1× TaqMan Gene Expression Master Mix and 1 μL of RT product used as the PCR template. The standard qPCR steps were as follows: 2 min at 50 °C and 10 min at 95 °C followed by 40 cycles of 15 s at 95 °C and 1 min at 60 °C. The threshold value (Ct) for each sample was set during the exponential phase, and the ΔΔ Ct method was used for data analysis. *Gapdh* was used as a reference gene.

### Western Blot Analysis

Cells were seeded on 6-well plates for western blot analysis. After 1, 3, 6, 24, or 48 h of exposure to 10 μM DEHP, western blot samples were collected. For immunoblotting, the cells were lysed in 100 μL of ice-cold lysis buffer containing 100 mM NaCl, 50 mM Tris HCl (pH 7.5), 0.5% Na-deoxycholate, 0.5% Nonidet NP-40, and 0.5% SDS. The lysates were then sonicated and clarified by centrifuging at 4 °C and 15,000×*g* for 20 min. The supernatant was collected and stored at − 80 °C until it was analyzed. The protein concentrations of the supernatants were determined using the Bradford method (Bradford 1976) with bovine serum albumin (BSA) as the standard. From the whole cell lysates, 20 μg of total protein were reconstituted in the appropriate amount of sample buffer, consisting of 125 mM Tris (pH 6.8), 4% SDS, 25% glycerol, 4 mM EDTA, 20 mM DTT, and 0.01% bromophenol blue. The samples were separated by 7.5% SDS-polyacrylamide gel electrophoresis in a Bio-Rad Mini-protean II Electrophoresis Cell. The protein was then transferred to nitrocellulose membranes using a Bio-Rad Mini Trans-Blot apparatus. Following the transfer, the membranes were washed and blocked with 5% dried milk and 0.2% Tween 20 in 0.02 M TBS for 2 h to prevent any nonspecific binding. The membranes were then incubated overnight with anti-AhR, anti-Cyp1a1, and anti-Cyp1b1 antibodies at a dilution of 1:200 in TBS/Tween at 4 °C. After incubation with primary antibody, the membranes were washed with TBS and 0.02% Tween 20 then incubated for 2 h with horseradish peroxidase-conjugated secondary antibodies diluted at 1:1000 in TBS/Tween. To control for the amount of protein that was loaded onto the gel, an anti-β-actin antibody diluted at 1:1000 in TBS/Tween (secondary antibody diluted at 1:5000 in TBS/Tween) was used. Signals were detected by chemiluminescence (ECL) using a Western Blotting Luminol Reagent and visualized with a FujiLas 4000 PhosphorImager. Immunoreactive band intensities were quantified by densitometry using an image analyzer with ImageJ 1.47v software (National Institute of Health, USA).

### Staining with Calcein AM

Calcein AM staining was performed to measure the intracellular esterase activity and to show cell morphology in neuron and glial cell cultures 24 h after an initial treatment with 10 μM of DEHP. This staining method was used to indicate metabolic activity and cell viability, according to a previously described protocol (Szychowski et al. 2015). Briefly, the cells grown on glass cover slips were then incubated in 4 μM calcein AM in PBS at 37 °C in an atmosphere of 5% CO_2_ for 10 min. Cells with light-green cytoplasm were identified as living cells using NIKON Eclipse 80i, (NIKON Instruments Inc., Melville, New York, USA) equipped with a camera with the BCAM Viewer© Basler AG software. A quantitative assessment of cell viability based on fluorescence measurement was performed as the separate experiment. The cells plated on 96-well plates were cultured in the presence of 10 μM of DEHP for 24 h. Then calcein AM solution was added to each well and incubated for 30 min at 37 °C. The measurement of the intracellular esterase activity was conducted with a fluorescence plate reader (Bio-Tek Instruments, Biokom) with 485 nm of excitation and 538 nm of emission wavelengths.

### Statistical Analysis

The data are presented as the mean ± standard deviation (SD) of four independent experiments. Each treatment was repeated eight times (*n* = 8) and run in triplicate; therefore, the total number of replicates was 24. The average of the quadruplicate samples was used for the statistical calculations. The data was analyzed using Multi-Mode Analysis software and was normalized to the fluorescence in a vehicle-treated control (% of control). The data were analyzed by one-way analysis of variance (ANOVA) followed by Tukey’s multiple comparison procedure. Differences between the control and experimental groups were marked probability (*p*) value as follows: ∗*p* < 0.05, ∗∗*p* < 0.01, and ∗∗∗*p* < 0.001.

## Results

### Reactive Oxygen Species Production

**Fig. 1:**
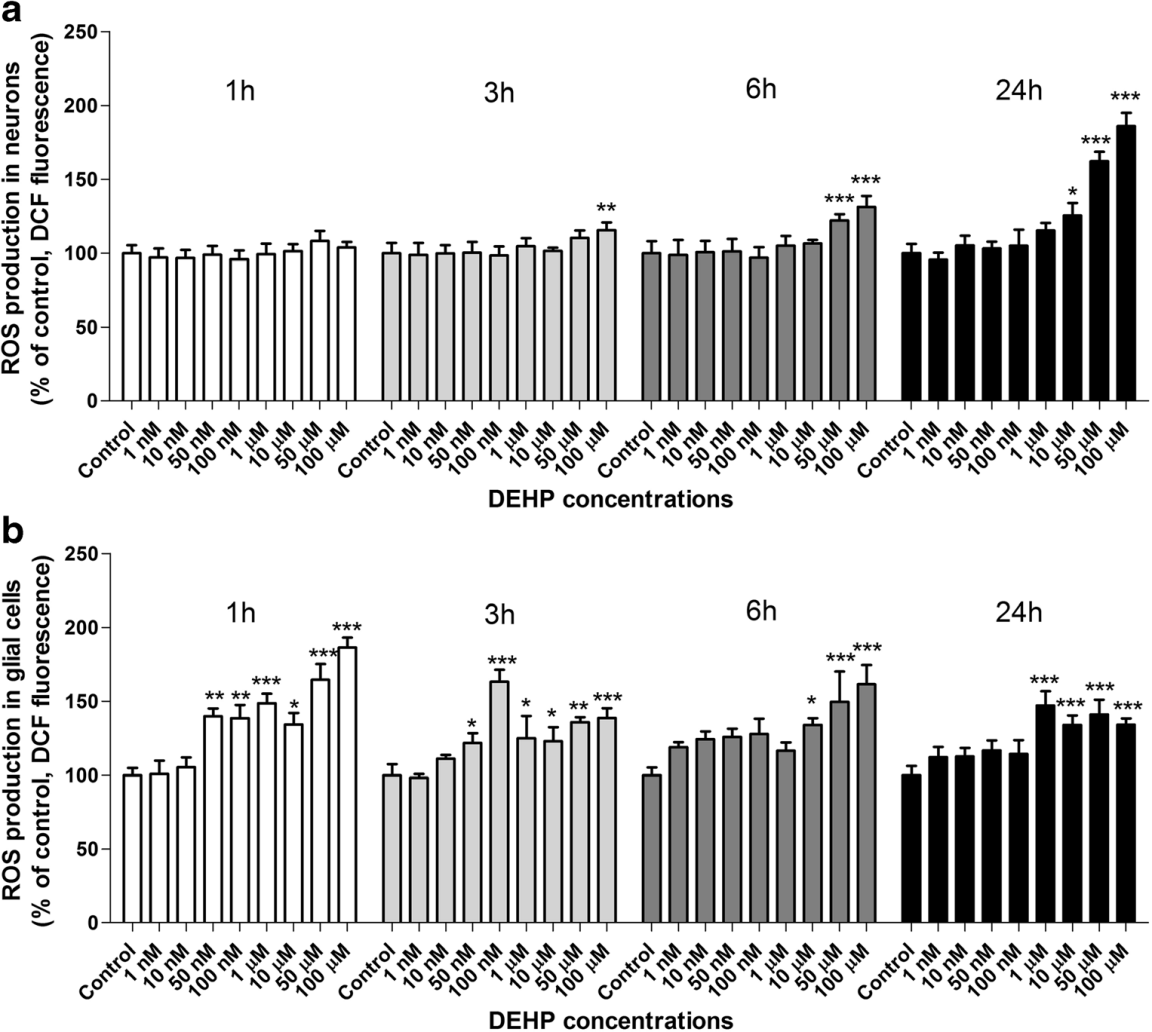
Effects on ROS production after 1, 3, 6, and 24 h of exposure to increasing DEHP concentrations in mouse primary neurons (**a**) and glial cells (**b**) in vitro. The data are expressed as the means ± SD of three independent experiments, each of which consisted of eight replicates per treatment group. ∗*p* < 0.05, ∗∗*p* < 0.01, and ∗∗∗*p* < 0.001 versus control cells

After neurons were exposed for 1 h to 1 nM–100 μM DEHP, any changes in ROS production were noted. After 3 h of exposure to DEHP, only the highest (100 μM) concentration increased ROS production by 15.55% compared with controls. After 6 h of exposure, 50 and 100 μM DEHP increased ROS production by 22.17 and 31.33%, respectively. In neurons exposed to DEHP for 24 h, we observed an increase in ROS production at concentrations of 10, 50, and 100 μM (increases of 25.50, 62.27, and 86.05%, respectively) (Fig. 1a).

Following 1 and 3 h of exposing glial cells to 1 nM–100 μM DEHP we observed an increase in ROS production from a range of 50 nM to 100 μM (increase from 21.82 to 64.58% compared with vehicle controls). However, after 6 h of exposure to DEHP only the high μM concentrations (10–100 μM) increased ROS production (increased by 33.95 to 62.64% compared with controls). After 24 h of exposure to DEHP, the ROS production increased in range from 1 to 100 μM (increases of 47.10 to 34.17% compared with vehicle controls) (Fig. 1b).

### EROD Activity

**Fig. 2:**
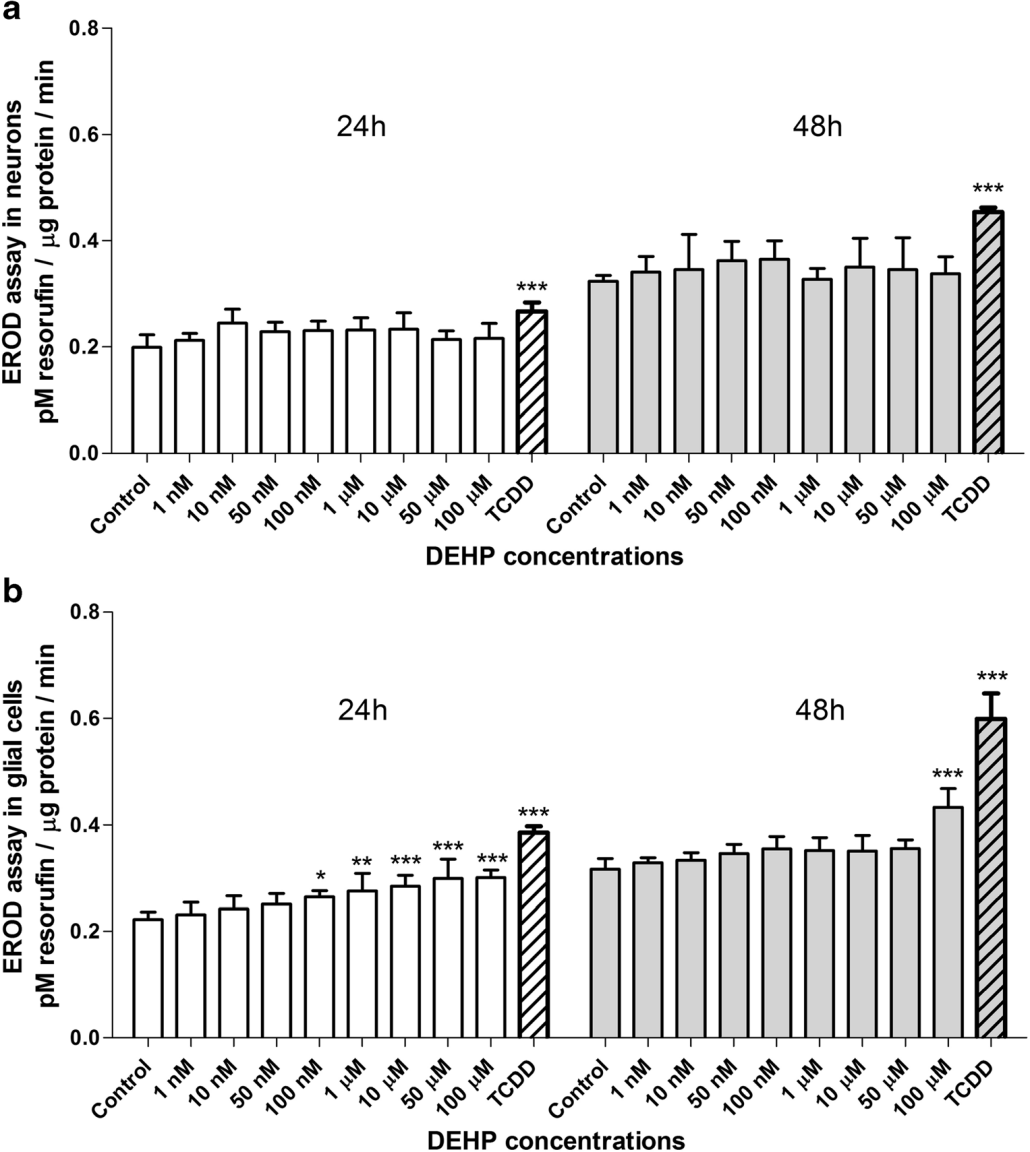
Effects on EROD activity after 24 and 48 h of exposure to increasing DEHP concentrations in mouse primary neurons (**a**) and glial cells (**b**) in vitro. The data are expressed as the means ± SD of three independent experiments, each of which consisted of eight replicates per treatment group. ∗*p* < 0.05, ∗∗*p* < 0.01, and ∗∗∗*p* < 0.001 versus control cells

After 24 and 48 h of exposing neurons to 1 nM–100 μM DEHP, any changes in EROD activity were noted. TCDD was used as a positive control and caused an increase in EROD activity after both 24 and 48 h (increases of 36.84 and 40.62%, respectively, compared with controls) (Fig. 2a).

In glial cells exposed to 1 nM–100 μM DEHP for 24 h, we observed an increase in EROD activity in a range of 100 nM to 100 μM (increases of 18.19 to 50.00% compared with vehicle controls). However, after 48 h of exposure, only the 100-μM DEHP increased EROD activity by 38.70%. TCDD strongly increased EROD activity after 24 and 48 h (increases of 50.00 and 72.72%, respectively, compared with controls) (Fig. 2b).

### Expression of *Ahr*, *Cyp1a1*, and *Cyp1b1* mRNA

**Fig. 3:**
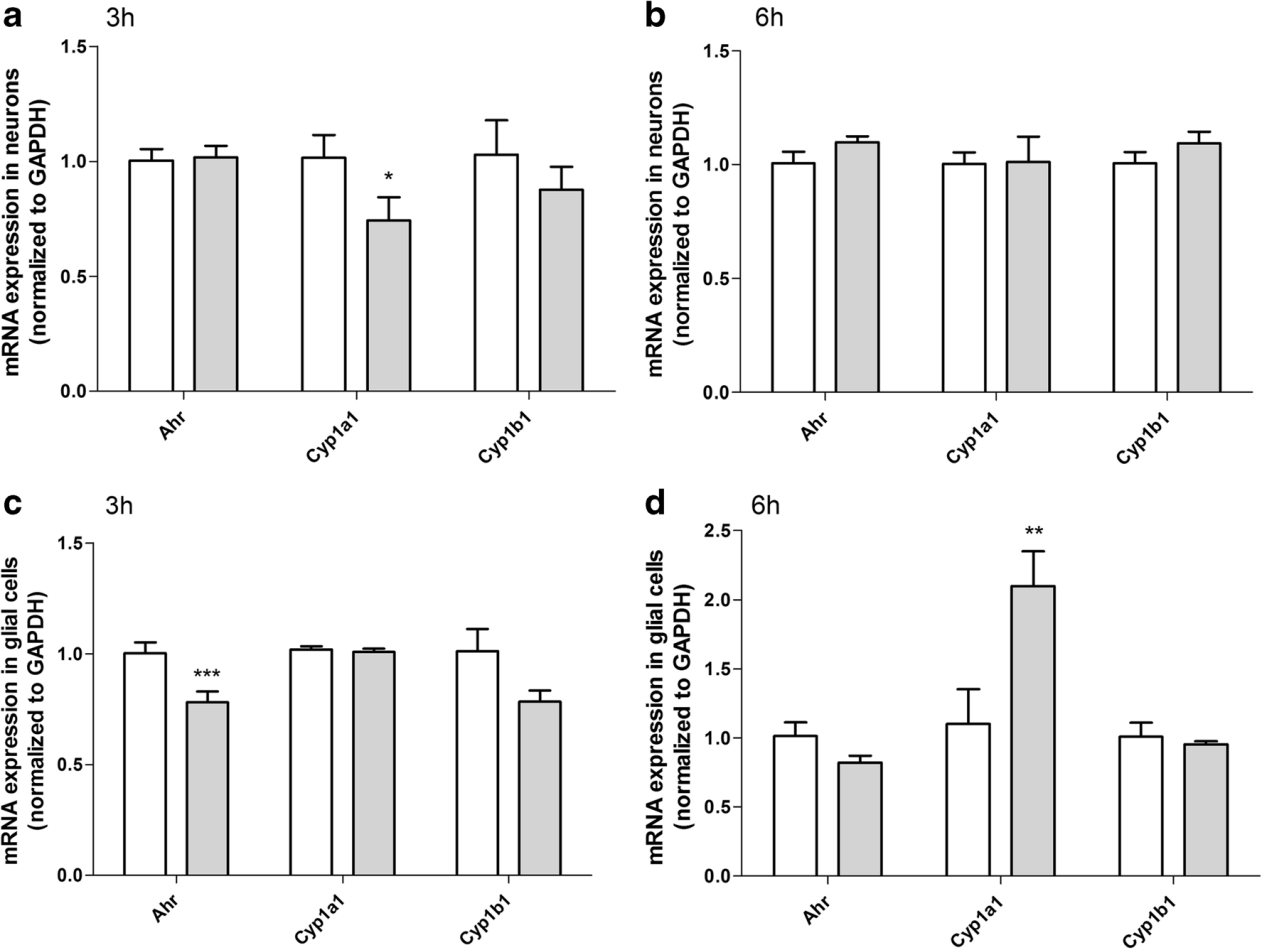
The effects of exposure to 10 μM DEHP on mRNA *ahr*, *cyp1a1*, and *cyp1b1* gene expression in mouse primary neurons after 3 h (**a**) and 6 h (**b**) and glial cells after 3 h (**c**) and 6 h (**d**) *in vitro*. The data are expressed as the means ± SD of three independent experiments, each of which consisted of eight replicates per treatment group. ∗*p* < 0.05, ∗∗*p* < 0.01, and ∗∗∗*p* < 0.001 versus control cells

After 3 h of exposure to 10 μM DEHP the neocortical neurons showed a decrease in expression of *Cyp1a1* mRNA by 25.57% compared with the vehicle control (Fig. 3a). However, in glial cells 10 μM DEHP decreased the expression of *Ahr* mRNA by 22.05% compared with vehicle controls (Fig. 3c).

After 6 h of exposure to 10 μM, DEHP neurons showed no change in gene expression (Fig. 3b). In contrast, after 6 h of exposure to 10 μM DEHP glial cells displayed an increase in *Cyp1a1* mRNA expression of 100.00% compared with vehicle controls (Fig. 3d).

### Expression of AhR, Cyp1a1, and Cyp1b1 Protein

**Fig. 4:**
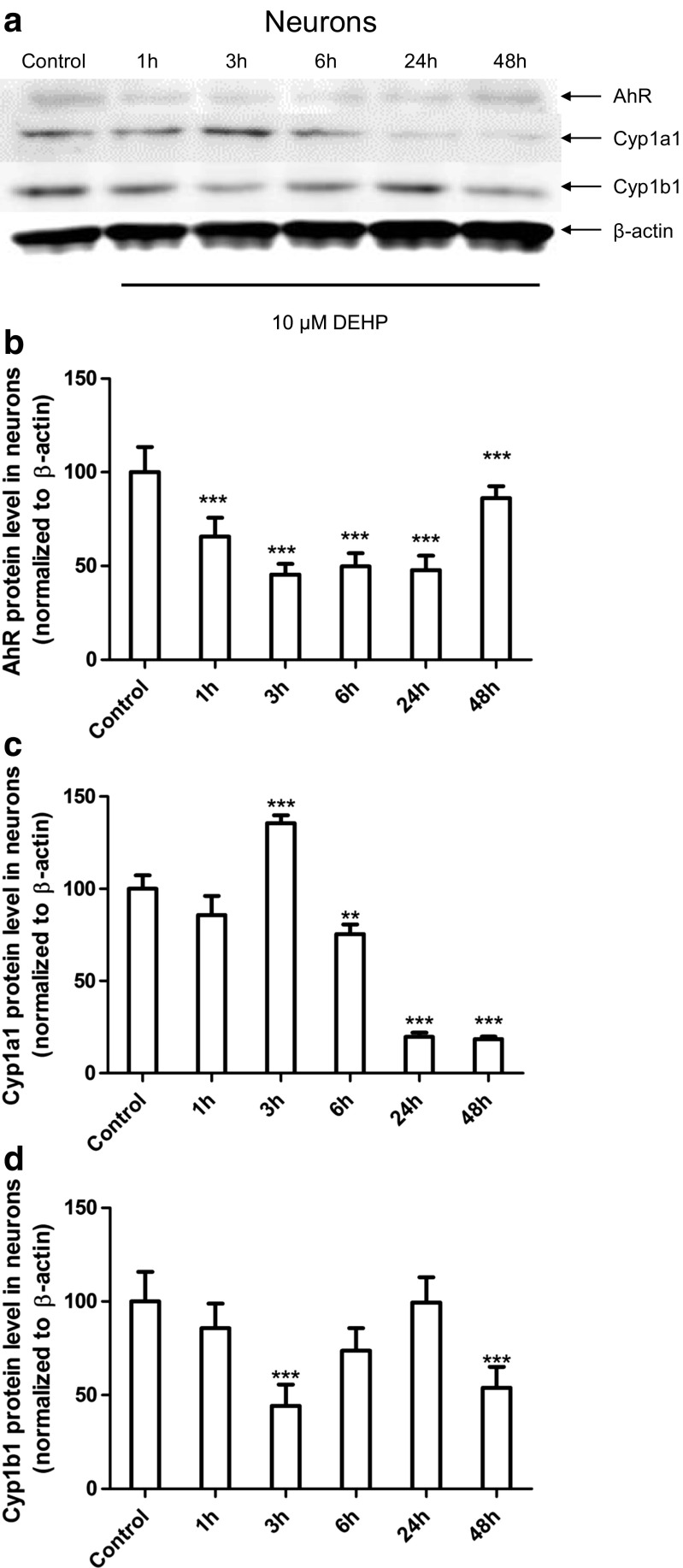
The effects of 10 μM DEHP on protein expression of Ahr, Cyp1a1, and Cyp1b1 after 1, 3, 6, 24, and 48 h in mouse primary neurons (**a**) in vitro. Protein bands were quantified by densitometry. The results are shown as the percentage of Ahr (**b**), Cyp1a1 (**c**), and Cyp1b1 (**d**) proteins relative to the control protein levels. Each column represents the mean ± SD of three independent experiments. The blots were stripped and reprobed with an anti-β-actin antibody to control for the amounts of protein loaded onto the gel. ∗∗*p* < 0.01 and ∗∗∗*p* < 0.001 versus the control

In neurons, immunoblot analyses quantified by densitometry demonstrated that 10 μM DEHP decreased AhR protein expression in all the time periods studied compared with controls (1, 3, 6, 24, and 48 h, decreased by 34.32.54.55, 50.14, 52.17, and 13.87%, respectively). After 3 h, an increase of 35.58% in Cyp1a1 protein expression was observed. However, after 6, 24, and 48 h, Cyp1a1 protein expression was significantly decreased by 22.54, 80.26, and 81.49%, respectively. Cyp1b1 protein expression was decreased after 3 and 48 h by 55.71 and 46.04%, respectively, compared with controls (Fig. 4).

**Fig. 5:**
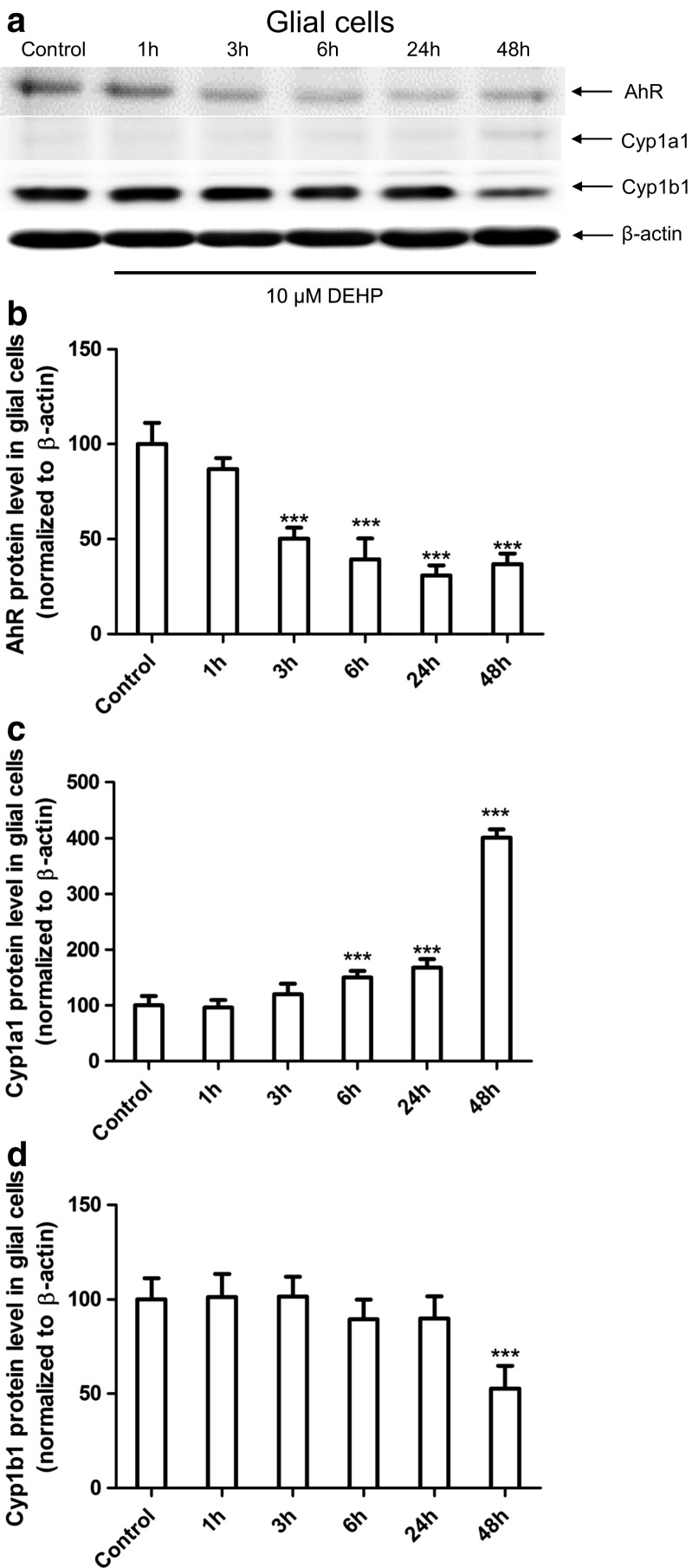
The effect of 10 μM DEHP on protein expression of Ahr, Cyp1a1, and Cyp1b1 after 1, 3, 6, 24, and 48 h in mouse primary glial cells (**a**) in vitro. Protein bands were quantified by densitometry. The results are shown as the percentage of Ahr (**b**), Cyp1a1 (**c**), and Cyp1b1 (**d**) proteins relative to the control protein levels. Each column represents the mean ± SD of three independent experiments. The blots were stripped and reprobed with an anti-β-actin antibody to control for the amounts of protein loaded onto the gel. ∗∗∗p < 0.001 versus the control

In glial cells, immunoblot analyses quantified by densitometry demonstrated that 10 μM DEHP decreased AhR protein expression after 3, 6, 24, and 48 h of exposure compared with controls (decreased by 49.87, 60.73, 69.25, and 63.24%, respectively). An increase in Cyp1a1 protein expression was observed after 6, 24, and 48 h (increased by 50.05, 68.01, and 301.21%, respectively). Cyp1b1 protein expression decreased by 47.38% after 48 h compared with the control (Fig. 5).

### Calcein AM Staining

**Fig. 6:**
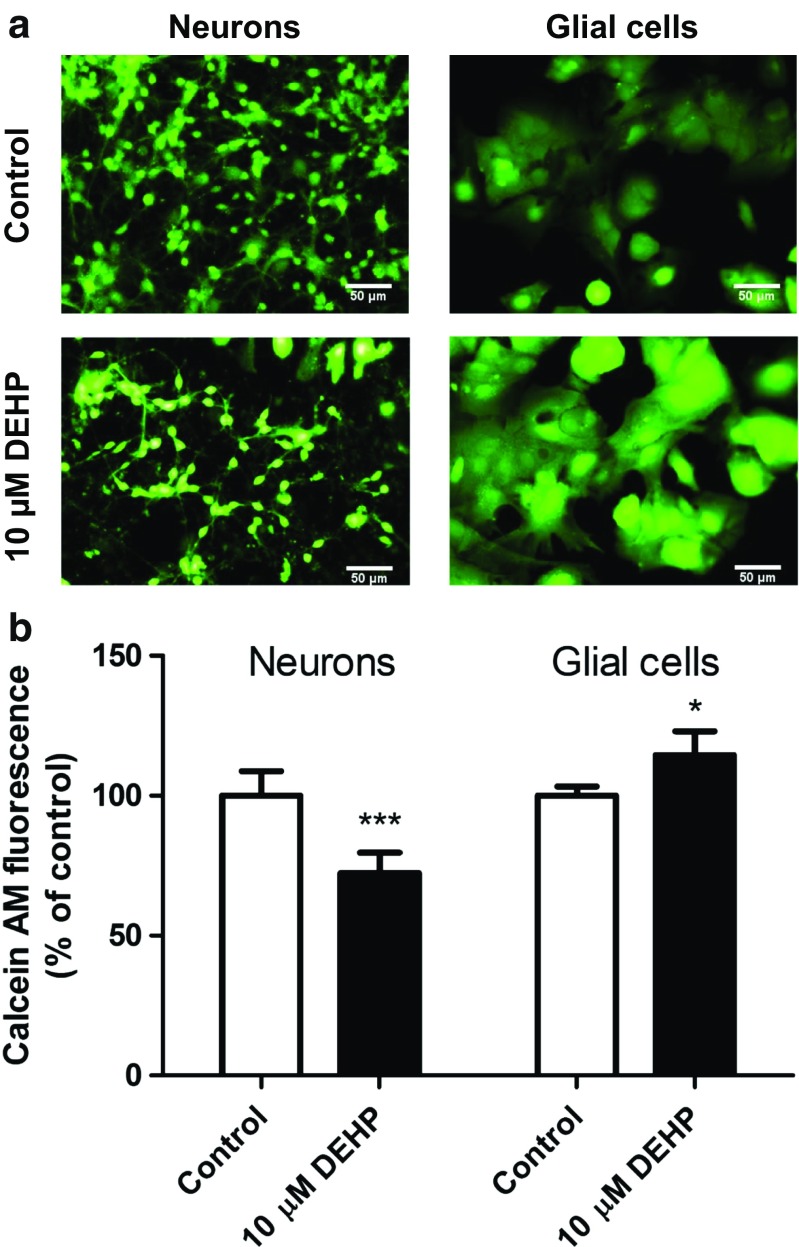
The effect of DEHP (10 μM) on mouse primary neocortical neurons and glial cells in vitro cultured at 7 DIV, examined 24 h post-treatment. Images of mouse primary neurons and glial cells cultures in vitro and stained with calcein AM. Cells with a light-colored cytoplasm were identified as viable cells (**a**); control neurons and neurons treated with 10 μM DEHP (left column). Control glial cells and glial cells treated with 10 μM DEHP (right column). The scale bar is at 50 μm. The results of calcein AM fluorescence measurement were performed using microplate fluorescence reader. The statistical data are expressed as the means ± SD of three independent experiments, each of which consisted of eight replicates per treatment group. ∗*p* < 0.05 and ∗∗∗*p* < 0.001 versus control cells (**b**)

In the control cultures, predominant healthy neurons as well as glial cells with the light green-fluorescence cytoplasm were presented. A reduction in living neurons and the increase in glial cells number were observed under the influence of DEHP (10 μM) (Fig. 6a). The fluorescence measurement confirmed that 10 μM of DEHP affected the viability/cells number. In neurons exposed to 10 μM DEHP for 24 h, we observed a decrease in calcein AM by 27.74%, compared with control. In glial cells exposed to 10 μM DEHP for 24 h, we observed an increase in calcein AM by 14.59%, compared with control (Fig. 6b).

## Discussion

Our experiments showed for the first time that DEHP stimulates ROS production in mouse neocortical cells, both in neuronal and glial cell cultures. In neocortical neurons, the highest concentration of DEHP (100 μM) increased ROS production after 3, 6, or 24 h of exposure. After the longer period of time of 24 h, the lower concentrations (10 and 50 μM) of DEHP also caused an increase in ROS production in mouse neurons. In primary glial cell cultures DEHP strongly stimulated ROS production after 1 and 3 h of exposure at a broad range of concentrations (from 50 nM to 100 μM). Nevertheless, after 6 and 24 h of exposure, similar to neurons, only the highest DEHP concentrations significantly increased ROS production in primary glial cells. DEHP-dependent ROS production is well-described in different culture models, mainly in relation to the reproductive system. Our data can be compared with the only relevant paper in which DEHP-dependent ROS production in in vitro neuron-astrocyte co-cultures has been studied (Wu et al. 2014). The data presented by Wu et al. (2014) demonstrated that even 1 nM DEHP caused a significant increase in ROS concentrations, indicating that neuronal-astrocyte co-cultures are more sensitive to DEHP than cerebral cell mono-cultures, as is also evident in our experiments. Additionally, in the mentioned study, astrocyte proliferation was initiated in response to DEHP, suggesting a mechanism of neuroprotection (Wu et al. 2014). Similar, in our experiments glial cells treated 10 μM DEHP and next stained by calcein AM increase in number wile neurons number decreased.

It is widely accepted that the stimulation of ROS may be an effect caused by increased expression and activity of Cyp1a1 (Kopf and Walker 2010). Because of this, we decided to study AhR, Cyp1a1, and Cyp1b1 expression to potentially elucidate DEHP’s mechanism of action. AhR and CYP1A1 are robustly expressed in neural progenitor cells (NPCs) and in various regions of the brain during critical periods of development both in neurons and glial cells (Tripathi et al. 2013; Dever et al. 2016). AhR and AhR-regulated CYP1A1 are known to mediate neuronal cell death in response to environmental pollutants as well as to be important regulators of metabolizing enzymes, detoxification, cell proliferation, differentiation, and inflammation (Hankinson 1995).

Our data showed for the first time that in neurons, *Ahr* mRNA expression does not change in response to DEHP, while AhR protein expression decreases. A similar trend was observed in regard to Cyp1a1 and Cyp1b1 mRNA and protein expression. Failure to induce Cyp1a1 was confirmed by the EROD assay. In primary glial cells, the decrease in AhR protein levels was accompanied by a decrease in *Ahr* mRNA expression. In these cells, both expression of Cyp1a1 mRNA and protein and Cyp1a1-related EROD activity significantly increased. *Cyp1b1* mRNA expression did not change significantly, and protein expression decreased only after 48 h of exposure to DEHP.

To date, phthalates have been accepted as exhibiting a weak potency as agonists of AhR (Mankidy et al. 2013). However, among the four phthalates studied (DEHP, diethyl phthalate (DEP), dibutyl phthalate (DBP), and benzyl butyl phthalate (BBP)), DEHP was the strongest inducer of AhR in *Rattus norvegicus* liver hepatoma (H4IIE) cells. According to different studies, ligand binding to AhR resulted in a decrease in the receptor protein level, which is an effect of proteolytic degradation of the complex (Song and Pollenz 2002; Filbrandt et al. 2004). These data support our hypothesis that DEHP-induced decreases in AhR protein levels in neurons and glial cells are caused by the activation of AhR.

According to Mankidy et al. (2013), DEHP targeted steroid biosynthesis pathways and stimulated production of estradiol (E2) with a simultaneous reduction in testosterone (T) concentrations. However, DEHP did not mimic E2 in an MCF-7-derived (MVLN) cell line as detected by bioluminescence transactivation assay (Mankidy et al. 2013). Nonetheless, in a study by Tanay Das et al. (2014) MCF-7 and MDA-MB-231 cell lines demonstrated that DEHP acts partially in an estrogen receptor alpha (ERα)-dependent manner (Tanay Das et al. 2014). Therefore, DEHP may stimulate E2 production and/or partially act as a disruptor of E2 signaling. It is widely accepted that estrogens and xenoestrogens can downregulate Cyp1a1 expression (Lai et al. 2004; Maradonna et al. 2004; Wójtowicz et al. 2011; Cocci et al. 2013), and a similar inhibitory effect by the estrogenic compound o,p′-DDT in Hepa cells has been reported by Jeong and Kim (2002). Because there are no data regarding DEHP action on CYP1A1, we can only compare our results with studies focused on other factors exhibiting estrogenic activity and downregulating CYP1A1, such as estradiol, estriol, 4-nonylphenol, methoxychlor, di-ortho-substituted polychlorinated biphenyls, and resveratrol (Ciolino et al. 1998; Jeong et al. 2001; Son et al. 2002; Han et al. 2007; Jablonska et al. 2011). These studies have shown that estrogens and estrogen-like compounds can inhibit CYP1A1 activity and/or CYP1A1 mRNA expression in Hepa cells, hepatocytes, and MCF7 (Ociepa-Zawal et al. 2007). It has been shown that E_2_ and benzophenone-2 (weak xenoestrogen) decrease in expression of *Ahr* mRNA in pituitary, thyroid, and uterus female Sprague–Dawley rats (Schlecht et al. 2004). Similar trend was observed by Lin et al. (2015b) where combined exposure of the mice to DEHP and Aroclor 1254 slightly but not significantly decrease expression of *AhR* mRNA expression in animal liver (Lin et al. 2015b). Furthermore, DEHP decrease in Cyp1a1 activity in rat liver (Seo et al. 2004). E2-mediated suppression of Cyp1a1 production is probably an effect caused by preventing the AhR complex from binding to the dioxin response element (DRE) (Lai et al. 2004). It is well documented that xenoestrogens can downregulate *aryl-hydrocarbon receptor nuclear translocator 2* (*ARNT2*) mRNA expression in human breast cancer cells through an ERα-dependent mechanism (Qin et al. 2011). Similarly, Jeong and Kim (2002) demonstrated an impairment of the dioxin-response element (DRE) being able to bind to DNA in o,p′-DDT-treated Hepa cells. Therefore, it appears that the inhibitory action of estrogenic compounds on CYP1A1 is universal across different tissues and may depend on AhR. However, Du et al. (2017) reported opposite results when they observed DEHP-induced cerebellar toxicity in *Coturnix japonica* by disrupting the CYP enzyme system homeostasis (Du et al. 2017). The authors showed an increase in both *AhR* and *Cyp1b1* mRNA expression*.* Similar results were observed in human immortalized granulosa cells (KGN). When the cells were exposed to 5 and 10 μM DEHP, there was an increase in *Ahr* mRNA expression but no effect on *CYP1B1* mRNA expression (Ernst et al. 2014). Hwang et al. (2005) demonstrated that humanized transgenic male mice with human CYP1B1 (hCYP1B1) given DEHP dose-dependently increased the activity and expression of mRNA and protein of hCYP1B1 (Hwang et al. 2005). However, it should be noted that transgenic mice co-expressing hCYP1B1 may not have the proper gene regulation sites preserved; therefore, the results may not be appropriate. Furthermore, several different mechanisms for AhR-ER crosstalk have been described to date and include competition for cofactors (ARNT) or competition for promoter binding sites (Kajta et al. 2007, 2009; Swedenborg and Pongratz 2010; Wójtowicz et al. 2017). Piechota et al. (2017) showed that neurons have higher levels of ERα than glial cells (Piechota et al. 2017). Therefore, it is our opinion that DEHP’s effects are probably tissue specific and are also dependent on the different levels of AhR and ERα receptors in the studied cells.

## Conclusion

Our results showed that DEHP phthalate increases ROS production in primary cell cultures of both neurons and glial cells. Our data showed for the first time that *AhR* mRNA expression in neurons does not change while protein expression of AhR decreases in response to DEHP. In primary glial cells, the decrease in AhR protein levels was accompanied by a decrease in *Ahr* mRNA expression. In neurons, DEHP decreased Cyp1a1 expression but did not change the activity of Cyp1a1, while in glial cells DEHP increased Cyp1a1 expression and activity. However, in both types of cells, DEHP decreased Cyp1b1 expression. We propose that the observed effects of DEHP action were probably cell-specific results.
